# Cross-sectional associations of maladaptive sleep-hygiene behaviors with athlete engagement among provincial- and municipal-level athletes in Sichuan, China

**DOI:** 10.3389/fpsyg.2026.1914692

**Published:** 2026-09-23

**Authors:** Yu Zhou, Yang Xu, Meilu Zhang, Huangyan Li

**Affiliations:** 1 College of Aviation Physical Education, Civil Aviation Flight University of China, Guanghan, Sichuan, China; 2 School of Humanities Education and Application, Sichuan Technology and Business University, Chengdu, Sichuan, China

**Keywords:** athlete engagement, cross-sectional study, emotion regulation, fatigue, PLS-SEM, sleep hygiene

## Abstract

**Purpose:**

Maladaptive sleep-hygiene behaviors are potentially modifiable daily practices, yet their relationship with the psychological state that sustains sport involvement remains unclear. This study examined cross-sectional associations among maladaptive sleep-hygiene behaviors (MSHB), multidimensional fatigue, difficulties in emotion regulation, and athlete engagement, including three model-based indirect associations.

**Methods:**

A cross-sectional questionnaire survey was completed by 317 adult athletes recruited from provincial- and municipal-level teams in Sichuan Province, China. Participants represented table tennis, badminton, tennis, basketball, volleyball, and football. The Sleep Hygiene Index, the Chinese Multidimensional Fatigue Inventory, the DERS-16, and the Athlete Engagement Questionnaire were administered. A reflective partial least squares structural equation model was estimated with 5,000 bootstrap resamples. Measurement quality, construct differentiation, common-method diagnostics, out-of-sample prediction, demographic adjustment, alternative cross-sectional specifications, and subgroup robustness were evaluated.

**Results:**

More frequent MSHB were associated with greater fatigue [*β* = 0.491, 95% CI (0.408, 0.573)], greater difficulties in emotion regulation [*β* = 0.178, 95% CI (0.079, 0.274)], and lower athlete engagement [*β* = −0.264, 95% CI (−0.370, −0.161)]. Fatigue was associated with greater difficulties in emotion regulation [*β* = 0.588, 95% CI (0.500, 0.673)] and lower engagement [*β* = −0.325, 95% CI (−0.446, −0.205)]; difficulties in emotion regulation were also associated with lower engagement [*β* = −0.232, 95% CI (−0.351, −0.108)]. The total indirect association was [*β* = −0.268, 95% CI (−0.338, −0.204)], and the model explained 47.2% of engagement variance. Demographic adjustment yielded comparable estimates. Reverse-order and parallel specifications fitted the cross-sectional covariance structure equally well.

**Conclusion:**

Within the sampled setting, more frequent MSHB co-occurred with greater fatigue, more difficulties in emotion regulation, and lower athlete engagement. Fatigue accounted for the largest specific indirect association. The single-occasion self-report design leaves actual sleep disturbance, temporal sequence, and causal effects unresolved.

## 1 Introduction

Sleep supports the physiological restoration, psychological well-being, cognitive functioning, and performance-related readiness required in competitive sport (Fullagar et al., 2015; Samuels, 2008; Walsh et al., 2021). Athlete sleep varies across age, sex, sport, competitive expertise, and season, and it is further shaped by early training, evening competition, travel, and accumulated workload (Anderson et al., 2024; Barba et al., 2025; Borghi et al., 2025; Nédélec et al., 2015; Vlahoyiannis et al., 2021). These influences make sleep relevant to everyday functioning in sport while also complicating the interpretation of any single sleep-related measure.

Sleep duration, timing, efficiency, night-to-night variability, perceived quality, insomnia symptoms, daytime sleepiness, and sleep-hygiene practices describe different aspects of sleep and recovery. The Sleep Hygiene Index (SHI) assesses behaviors such as inconsistent sleep–wake schedules, prolonged napping, stimulating activities near bedtime, substance-related habits, and wakeful activity in bed (Mastin et al., 2006). Its score reflects the frequency of practices that may interfere with sleep. Objective sleep duration, efficiency, circadian timing, and nightly variability remain outside the construct measured by the SHI (Seun-Fadipe et al., 2018; Vatne et al., 2025). The predictor in this study is therefore termed maladaptive sleep-hygiene behaviors (MSHB).

Athlete engagement is a positive sport-related state characterized by confidence, dedication, vigor, and enthusiasm (Lonsdale et al., 2007; Martins et al., 2014). It reflects energized psychological involvement in training and competition rather than attendance or competitive performance. Engagement has been linked to motivational climate, passion, and other social or motivational conditions (Curran et al., 2015; Schellenberg and Lötscher, 2024). Recovery-related conditions may provide an additional source of variation because workload, perceived recovery, fatigue, and psychological state change together across training periods (Curzi et al., 2024; Hamlin et al., 2021; Long et al., 2024).

The conceptual model connects MSHB with athlete engagement directly and through two psychological correlates. Fatigue represents a multidimensional subjective state involving physical and mental fatigue, reduced activity, and reduced motivation (Enoka and Duchateau, 2016; Knicker et al., 2011). Sleepiness refers more narrowly to the propensity to fall asleep; it was not assessed. Difficulties in emotion regulation concern problems accepting emotional responses, maintaining goal-directed behavior under distress, controlling impulses, accessing effective strategies, and understanding emotional states (Bjureberg et al., 2016; Kökönyei et al., 2014). Both constructs are relevant in sport settings marked by repeated evaluation, setbacks, selection uncertainty, and interpersonal demands (Doron et al., 2025; Kucharski et al., 2018; Robazza et al., 2023).

Fatigue was placed before difficulties in emotion regulation in the hypothesized ordering because emotion regulation draws on attentional and motivational resources that may be less available when fatigue is elevated. Other orderings remain plausible: persistent regulation difficulties may intensify perceived fatigue, lower engagement may coincide with poorer self-care, and common training or competition demands may influence all four constructs. The analysis consequently treats the model as a cross-sectional associational structure. Its contribution lies in extending athlete-sleep research toward modifiable behavioral practices and athlete engagement while evaluating whether the estimated associations remain stable across measurement, predictive, covariate, alternative-order, and subgroup analyses.

## 2 Conceptual framework and hypotheses

### 2.1 Maladaptive sleep-hygiene behaviors and athlete engagement

Sleep-hygiene behaviors encompass routines and environmental practices that shape sleep opportunity. Within the SHI, these behaviors include inconsistent bed and wake times, extended naps, stimulating activities near bedtime, substance use that may affect sleep, and cognitive or emotional activity in bed (Mastin et al., 2006). Competitive schedules may make such practices difficult to regulate because training times, competition, travel, recovery treatments, media use, and academic or social demands compete for limited time. Chronotype, sport discipline, and training structure further influence sleep behavior across athletes (Borghi et al., 2025; Pullinger et al., 2025; Vlahoyiannis et al., 2021).

A high SHI score indicates frequent engagement in behaviors with the potential to interfere with sleep. The effect of a given behavior depends on its timing, duration, individual susceptibility, and the surrounding training schedule. Training-session type and in-season demands are themselves associated with changes in sleep (Barba et al., 2025; Grewal et al., 2024). Behavioral assessment is therefore most informative when interpreted alongside diaries, actigraphy, or other objective sleep indicators (Halson, 2019; Walsh et al., 2021).

A contemporaneous association with athlete engagement is plausible because engagement contains energetic and affective components. Confidence concerns perceived capacity to attain sport goals, dedication reflects sustained commitment, vigor represents physical and mental energy, and enthusiasm captures positive activation (Lonsdale et al., 2007). Athletes reporting more maladaptive sleep-hygiene practices may also report lower readiness, less stable attention, or reduced vitality, experiences that align with lower vigor and enthusiasm. The relationship is expected to remain probabilistic because engagement is also shaped by coaching, motivational climate, passion, role security, and broader life demands (Curran et al., 2015; Schellenberg and Lötscher, 2024).

### 2.2 Fatigue and difficulties in emotion regulation as indirect correlates

The Chinese Multidimensional Fatigue Inventory represents fatigue through physical fatigue, mental fatigue, reduced activity, and reduced motivation (Miao et al., 2008; Smets et al., 1995). This broad subjective state may arise from training load, illness, injury, psychological strain, travel, nutrition, and incomplete recovery (Enoka and Duchateau, 2016; Halson, 2019; Knicker et al., 2011). Sleep restriction and disrupted recovery are commonly associated with fatigue and perceived exertion, although the magnitude of these relationships varies across athlete populations and training contexts (Campbell et al., 2021; Fullagar et al., 2015; Kong et al., 2025).

MSHB may covary with fatigue through several routes. Some behaviors can reduce sleep opportunity or increase pre-sleep activation; high training or competitive demands can simultaneously disturb routines and elevate fatigue; and common stressors can influence both reports. In-season monitoring studies show that sleep, workload, and well-being often change together rather than independently (Grewal et al., 2024; Long et al., 2024). Objective sleep was not assessed in the current survey, leaving the intermediate link between sleep-hygiene behavior and actual sleep disturbance unobserved.

Fatigue is also relevant to athlete engagement. Training participation requires energy, concentration, and willingness to persist through discomfort, repetition, and evaluation. Greater fatigue may correspond with lower vigor and enthusiasm, while reduced activity and motivation may accompany weaker dedication. Athlete-reported fatigue and recovery indicators have been associated with later performance-related behavior in team sports, although findings are not uniform (Sansone et al., 2023). Psychological fatigue has also been linked with athlete engagement in speed skating (Wang et al., 2025).

Difficulties in emotion regulation capture nonacceptance, impaired goal-directed behavior under distress, impulse-control problems, restricted access to effective strategies, and limited emotional clarity (Bjureberg et al., 2016). These difficulties are salient in sport because athletes must manage mistakes, interpersonal tension, selection uncertainty, and changing performance demands while remaining goal directed. Regulation strategies vary across sport contexts, and cognitive reappraisal is related to athletes’ emotional experiences and psychological needs (Kucharski et al., 2018; Robazza et al., 2023). Coping effectiveness and engagement also fluctuate together during intensive training (Doron et al., 2025).

Sleep loss and sleep-related problems have been associated with altered cognitive control, stress responses, and emotional functioning (Song et al., 2025; Vitale et al., 2021). Athletes reporting more MSHB may therefore report greater difficulty regulating emotions, while regulation difficulties may correspond with lower confidence, dedication, and enthusiasm under pressure. The SHI does not identify whether sleep loss occurred, so the proposed indirect association remains anchored to the measured behavioral and psychological variables.

The measures also contain adjacent themes. Reduced motivation and activity are fatigue domains, vigor and dedication are engagement domains, and difficulty maintaining goal-directed behavior is part of the DERS-16. Such content proximity can increase correlations even when the broader constructs remain distinguishable. The measurement analysis therefore examined HTMT confidence intervals and compared the four-construct model with models that combined fatigue and difficulties in emotion regulation.

### 2.3 Model ordering and hypotheses

The hypothesized serial ordering places fatigue before difficulties in emotion regulation. Elevated fatigue may coincide with reduced attentional control, lower motivational resources, and greater perceived effort, making goal-directed regulation under distress more difficult (Knicker et al., 2011; Kucharski et al., 2018). Difficulties in emotion regulation may then correspond with lower engagement. The reverse ordering is equally credible, and common workload or competitive pressures may affect both constructs at the same time (Curzi et al., 2024; Long et al., 2024). The serial model represents one theory-organized specification of the cross-sectional covariance structure; reverse-order and parallel specifications were examined as sensitivity analyses.

The hypotheses concern direct and model-based indirect associations measured at the same occasion:

*H1:* More frequent maladaptive sleep-hygiene behaviors are negatively associated with athlete engagement.

*H2:* Maladaptive sleep-hygiene behaviors show a negative indirect association with athlete engagement through fatigue.

*H3:* Maladaptive sleep-hygiene behaviors show a negative indirect association with athlete engagement through difficulties in emotion regulation.

*H4:* Maladaptive sleep-hygiene behaviors show a negative serial indirect association with athlete engagement through fatigue and difficulties in emotion regulation in the hypothesized model order.

Figure 1 presents the direct paths and the three model-based indirect associations. The arrows denote the statistical specification.

**Figure 1:**
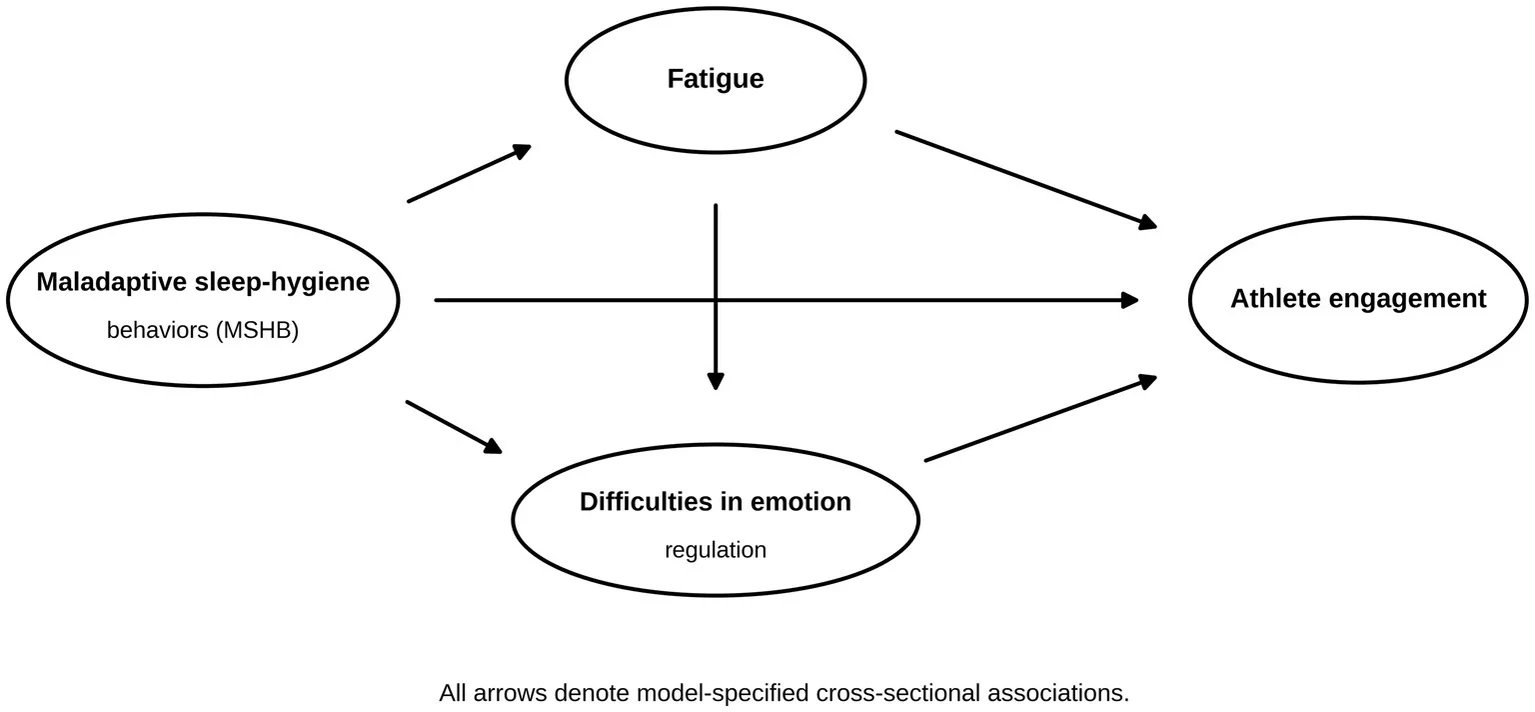
Hypothesized cross-sectional associational path model. Single-headed arrows indicate model-specified associations. Temporal and causal direction cannot be inferred from the cross-sectional design.

## 3 Materials and methods

### 3.1 Design, participants, and procedure

A cross-sectional questionnaire survey was conducted from September 15 to November 1, 2025, during the autumn training period. The Ethics Committee of the Civil Aviation Flight University of China approved the protocol on December 26, 2024 (Approval No. 20240014), before recruitment and data collection. All participants were adults and provided written informed consent. The procedures followed the Declaration of Helsinki.

Participants were recruited through nonprobability convenience sampling during scheduled visits to provincial- and municipal-level sports teams in Sichuan Province, China. Within the Chinese sport system, provincial and municipal describe the administrative affiliation of teams to sport authorities at those levels. These labels do not provide an internationally harmonized elite classification or information about professional employment. The sample represented table tennis, badminton, tennis, basketball, volleyball, and football. The study size reflected the athletes accessible during the scheduled survey visits.

Trained investigators administered paper questionnaires at the athletes’ training venues using standardized oral and written instructions. Participation was anonymous and voluntary. Athletes completed the survey individually and returned it directly to the investigators. They were informed that responses would be analyzed in aggregate and would have no bearing on team selection, training evaluation, or competition opportunities. Coaches and team managers had no access to individual responses. The measures appeared in separate sections, and demographic questions were separated from the focal constructs to reduce evaluation apprehension and demand-related responding (Podsakoff et al., 2003).

Four hundred questionnaires were distributed and 364 were returned, yielding a return rate of 91.00%. A returned form was excluded when more than 10% of responses were missing or multiply marked, the same response option appeared on at least 80% of items, logically incompatible responses occurred across reverse-keyed or semantically overlapping items, or the form was illegible or otherwise unscorable. Forty-seven forms met at least one criterion. The criteria overlapped, and mutually exclusive criterion-specific counts were unavailable. The analytic sample comprised 317 questionnaires, equivalent to 87.09% of returned forms and 79.25% of distributed forms. The final dataset contained no missing values.

Age was recorded in four categories: 18–24, 25–29, 30–34, and 35 years or older. The median category was 25–29 years; exact age values were unavailable for calculation of a numerical mean, standard deviation, and range. The survey also recorded sex, education, sport, years of sport experience, and competitive level in China’s athlete technical-grade system. Professional, amateur, or student-athlete status; training frequency and volume; training time of day; competition phase; selection or starting-position pressure; academic demands; injury and health status; nutrition; travel; ambient temperature; team and organization identifiers; and individual survey dates were not recorded. Sampling records did not retain the number of participating teams or the denominator of all eligible athletes. Team-level clustering could not be estimated. Available demographic variables and sport were included in sensitivity analyses. Participant characteristics are summarized in Table 1.

**Table 1:** Demographic characteristics of participants (*N* = 317).

Variable	Category	*n*	%
Sex	Male	161	50.79
	Female	156	49.21
Age	18–24 years	85	26.81
	25–29 years	99	31.23
	30–34 years	69	21.77
	≥35 years	64	20.19
Education level	Associate degree or below	98	30.91
	Bachelor’s degree	109	34.38
	Master’s degree or above	110	34.70
Sport	Table tennis	64	20.19
	Badminton	46	14.51
	Tennis	44	13.88
	Basketball	59	18.61
	Volleyball	56	17.67
	Football	48	15.14
Sport experience	<5 years	91	28.71
	5–10 years	80	25.24
	>10 to 15 years	73	23.03
	>15 years	73	23.03
Competitive level	National second-class athlete or below	105	33.12
	National first-class athlete	121	38.17
	National-level athlete or above	91	28.71

Age was collected in categories; the median category was 25–29 years. Competitive-level categories refer to China’s athlete technical-grade system and denote progressively higher competitive credentials; they do not establish professional employment or an internationally standardized elite classification.

### 3.2 Measures and language adaptation

All focal constructs were assessed by self-report using five-point response formats. The published Chinese Multidimensional Fatigue Inventory was used. Chinese versions of the SHI, DERS-16, and AEQ were prepared through forward translation, reconciliation, independent back-translation, and review by bilingual researchers with sport-psychology expertise. Adult athletes checked the translated wording for clarity and comprehensibility before the main survey. The procedure followed established back-translation principles (Brislin, 1970). Internal consistency, convergent and discriminant validity, higher-order loadings, and subgroup measurement invariance were evaluated in the study sample. Domain-level descriptive statistics and internal-consistency estimates for the multidimensional measures are reported in Supplementary Table S1.

#### 3.2.1 Maladaptive sleep-hygiene behaviors

The 13-item SHI assesses the frequency of behaviors that may interfere with sleep (Mastin et al., 2006). Content includes inconsistent sleep–wake schedules, prolonged naps, stimulating activity near bedtime, substance-related practices, and wakeful activity in bed. Responses ranged from 1 (never) to 5 (always). The mean of the 13 items was used, with higher scores indicating more frequent MSHB. The SHI was specified as a unidimensional reflective construct and showed Cronbach’s α = 0.950. Previous studies have supported its use as a behavioral sleep-hygiene screening measure (Seun-Fadipe et al., 2018). The score was interpreted as behavioral frequency; objective sleep duration, regularity, quality, insomnia, circadian disruption, and sleep efficiency were outside its measurement scope.

#### 3.2.2 Fatigue

The 20-item Chinese Multidimensional Fatigue Inventory was adapted by Miao et al. (2008) from the original MFI (Smets et al., 1995). It assesses physical fatigue (10 items), mental fatigue (4 items), reduced activity (3 items), and reduced motivation (3 items) over the preceding two weeks. Responses ranged from 1 (not true) to 5 (true). Item keying followed the administered Chinese questionnaire, and higher domain scores indicated greater fatigue. The item-to-variable crosswalk, domain membership, and keying rules are documented in the accompanying Data and Code archive. Domain means served as reflective indicators of the higher-order fatigue construct. Cronbach’s α values were 0.941 for physical fatigue, 0.851 for mental fatigue, 0.822 for reduced activity, and 0.773 for reduced motivation. The scale captures multidimensional fatigue; daytime sleepiness was outside the assessed construct.

#### 3.2.3 Difficulties in emotion regulation

The DERS-16 contains 16 items covering nonacceptance of emotional responses (3 items), difficulty maintaining goal-directed behavior under distress (3 items), impulse-control difficulties (3 items), limited access to effective strategies (5 items), and lack of emotional clarity (2 items; Bjureberg et al., 2016). Responses ranged from 1 (almost never) to 5 (almost always). Domain means were calculated and averaged with equal weight, with higher scores indicating greater difficulties. Domain α values ranged from 0.768 to 0.863. The five domain scores served as reflective indicators of the higher-order construct. An item-weighted score produced the same substantive pattern and correlated 0.993 with the domain-weighted score.

#### 3.2.4 Athlete engagement

The 16-item AEQ assesses confidence, dedication, vigor, and enthusiasm, with four items in each domain (Lonsdale et al., 2007). Its four-dimensional structure has also been supported in a Portuguese sport setting (Martins et al., 2014). Responses ranged from 1 (strongly disagree) to 5 (strongly agree). Domain means were averaged, with higher scores indicating stronger engagement. Domain α values ranged from 0.791 to 0.850. The four domain scores served as reflective indicators of the higher-order engagement construct. The questionnaire captures positive psychological involvement in sport; competitive performance was not assessed.

### 3.3 Statistical analysis

Data screening and descriptive analyses were performed in IBM SPSS Statistics 27. The complete analytic dataset required no imputation. Scale means were treated as continuous variables, and categorical demographic variables were represented by dummy indicators in sensitivity models. Descriptive means were reported without qualitative labels because validated interpretive cutoffs were unavailable.

PLS-SEM was selected for the prespecified higher-order model because the analytical objective combined explanation of engagement variance with out-of-sample prediction (Hair et al., 2019; Hair et al., 2021). The primary analysis was conducted in R 4.5.3 with cSEM 0.6.1. Traditional PLS-PM used path weighting, Mode A reflective measurement, a maximum of 300 iterations, a convergence tolerance as 10^–7^, and no disattenuation. The 13 SHI items represented MSHB. The four MFI domain scores, five DERS-16 domain scores, and four AEQ domain scores were calculated from their items before model estimation and used as reflective indicators of the corresponding higher-order constructs. A reflective-reflective specification was adopted because each set of correlated domains was treated as manifestations of a broader construct (Sarstedt et al., 2019).

Measurement evaluation included outer loadings, Cronbach’s α, composite reliability, average variance extracted (AVE), the Fornell-Larcker criterion, and heterotrait-monotrait ratios (HTMT). Two-sided 95% percentile confidence intervals for HTMT used 5,000 bootstrap resamples (Henseler et al., 2015). Robust maximum-likelihood (MLR) confirmatory factor analysis in Mplus 8.3 compared the four-construct model with a three-factor model that combined fatigue and difficulties in emotion regulation and with a one-factor model. Model reporting included χ^2^, CFI, TLI, RMSEA with 90% confidence limits, and SRMR. Nested-model comparisons used Satorra–Bentler scaled chi-square difference tests calculated from the model-specific MLR scaling correction factors.

Common-method risk was examined through procedural safeguards and three statistical diagnostics. Harman’s unrotated principal-component analysis used all 65 item variables. A one-factor confirmatory model was evaluated using the 26 indicators in the higher-order measurement model. Full-collinearity variance-inflation factors provided an additional diagnostic (Kock, 2015). The results were interpreted as sensitivity evidence; same-occasion self-report remained susceptible to shared-method variance (Podsakoff et al., 2003).

Structural evaluation included standardized path coefficients, bootstrap standard errors, two-sided percentile 95% confidence intervals, structural VIF, R^2^, and f^2^. Five thousand bootstrap resamples were generated with seed 20,260,810. Specific indirect associations were estimated through fatigue, through difficulties in emotion regulation, and through their model-specified sequence. Their sum, the direct association, and the total association were reported. Bootstrap contrasts compared the three specific indirect estimates.

Out-of-sample prediction was examined with PLSpredict using 10-fold cross-validation repeated 10 times and linear regression as the benchmark (Shmueli et al., 2019). A core covariate model adjusted for sex, age category, sport, and competitive level; an extended model also adjusted for education and years of sport experience. Alternative specifications reversed the order of fatigue and difficulties in emotion regulation or treated them as correlated parallel mediators. Measurement invariance of composites and PLS multigroup comparisons were evaluated across sex and broad sport type—individual racket/net sports versus team ball sports—using 5,000 permutations or resamples (Henseler et al., 2016). Reporting follows STROBE recommendations for cross-sectional observational studies (von Elm et al., 2007). A score-weighting sensitivity analysis compared the equal-domain DERS-16 score with the mean of all 16 items.

## 4 Results

### 4.1 Descriptive results, measurement quality, and common-method diagnostics

Descriptive statistics and measurement-quality indices are shown in Table 2. Mean scores were 2.48 (SD = 0.95) for MSHB, 2.55 (SD = 0.80) for fatigue, 2.59 (SD = 0.79) for difficulties in emotion regulation, and 3.52 (SD = 0.71) for athlete engagement. Cronbach’s α ranged from 0.781 to 0.950, composite reliability from 0.862 to 0.956, and AVE from 0.609 to 0.686. All 26 outer loadings were statistically different from zero and ranged from 0.751 to 0.862. Indicator-specific outer loadings and bootstrap confidence intervals are reported in Supplementary Table S2.

**Table 2:** Descriptive statistics, reliability, and convergent validity (*N* = 317).

Construct	M	SD	α	CR	AVE	Loading range
Maladaptive sleep-hygiene behaviors	2.48	0.95	0.950	0.956	0.628	0.751–0.855
Fatigue	2.55	0.80	0.848	0.897	0.686	0.802–0.862
Difficulties in emotion regulation	2.59	0.79	0.842	0.889	0.617	0.767–0.817
Athlete engagement	3.52	0.71	0.781	0.862	0.609	0.761–0.806

*α* = Cronbach’s alpha; CR, composite reliability; AVE, average variance extracted. Reliability and loading ranges refer to the indicators used at the higher-order stage.

The zero-order correlations followed the expected directions (Table 3). MSHB correlated positively with fatigue (*r* = 0.489) and difficulties in emotion regulation (*r* = 0.462) and negatively with athlete engagement (*r* = −0.528). Fatigue correlated positively with difficulties in emotion regulation (*r* = 0.673) and negatively with engagement (*r* = −0.608). Difficulties in emotion regulation correlated negatively with engagement (*r* = −0.571). All six correlations had *p* < 0.001.

**Table 3:** Correlations and Fornell-Larcker values (*N* = 317).

Variable	1	2	3	4
1. Maladaptive sleep-hygiene behaviors	0.792			
2. Fatigue	0.489***	0.828		
3. Difficulties in emotion regulation	0.462***	0.673***	0.785	
4. Athlete engagement	−0.528***	−0.608***	−0.571***	0.780

Diagonal values are square roots of AVE; off-diagonal values are Pearson correlations among the composite scores used for descriptive analysis. ***p < 0.001.

HTMT estimates and bootstrap confidence intervals appear in Table 4. All point estimates were below 0.80, and every 95% upper confidence limit was below 0.90. The fatigue–difficulties-in-emotion-regulation pair had the largest estimate, HTMT = 0.794, 95% CI [0.725, 0.853]. Its upper limit was close to the stricter 0.85 guideline, supporting examination of alternative factor structures.

**Table 4:** HTMT estimates and 95% bootstrap confidence intervals.

Construct pair	HTMT	95% CI
Fatigue–MSHB	0.545	[0.450, 0.632]
Difficulties in emotion regulation–MSHB	0.517	[0.415, 0.609]
Athlete engagement–MSHB	0.613	[0.514, 0.704]
Difficulties in emotion regulation–fatigue	0.794	[0.725, 0.853]
Athlete engagement–fatigue	0.747	[0.659, 0.825]
Athlete engagement–difficulties in emotion regulation	0.700	[0.605, 0.786]

MSHB, maladaptive sleep-hygiene behaviors. Confidence intervals used 5,000 percentile bootstrap resamples.

The four-factor confirmatory model fitted the data well, robust χ^2^(293) = 318.67, *p* = 0.145, CFI = 0.994, TLI = 0.994, RMSEA = 0.017, 90% CI [0.000, 0.028], and SRMR = 0.030 (Table 5). The model combining fatigue with difficulties in emotion regulation fitted less well, robust χ^2^(296) = 413.87, CFI = 0.974, TLI = 0.971, RMSEA = 0.035, 90% CI [0.027, 0.043], and SRMR = 0.034; scaled Δχ^2^(3) = 85.53, *p* < 0.001. The one-factor model also fitted substantially worse, robust χ^2^(299) = 1335.53, CFI = 0.767, TLI = 0.747, RMSEA = 0.105, 90% CI [0.099, 0.110], and SRMR = 0.105; scaled Δχ^2^(6) = 1013.94, *p* < 0.001. The factor comparisons and HTMT results supported empirical differentiation among the four constructs while retaining the substantive proximity between fatigue and difficulties in emotion regulation.

**Table 5:** Robust maximum-likelihood confirmatory factor analysis comparisons.

Model	Robust χ^2^ (df)	CFI	TLI	RMSEA [90% CI]	SRMR	Scaled Δχ^2^ vs four-factor
Four factors	318.67 (293)	0.994	0.994	0.017 [0.000, 0.028]	0.030	—
Fatigue and regulation difficulties combined	413.87 (296)	0.974	0.971	0.035 [0.027, 0.043]	0.034	85.53 (3)***
One factor	1335.53 (299)	0.767	0.747	0.105 [0.099, 0.110]	0.105	1013.94 (6)***

The four-factor model was the reference. Scaled Δχ^2^ values are Satorra–Bentler difference tests based on model-specific MLR scaling correction factors. ***p < 0.001.

Harman’s analysis of the 65 item variables produced 12 components with eigenvalues above 1. The first component accounted for 32.54% of total variance, and the retained components accounted for 66.73%. Full-collinearity VIF values ranged from 1.511 to 2.181. No dominant single-factor pattern was evident, although shared-method inflation remained plausible. Complete common-method diagnostic results are presented in Supplementary Table S3. The equal-domain and item-weighted DERS-16 scores correlated 0.993, and the largest absolute difference between their standardized composite-score path estimates was 0.009 (Supplementary Table S4).

### 4.2 Structural and indirect associations

The primary PLS model had SRMR = 0.044, and structural VIF values ranged from 1.000 to 1.982. More frequent MSHB were associated with greater fatigue, *β* = 0.491, 95% CI [0.408, 0.573], greater difficulties in emotion regulation, *β* = 0.178, 95% CI [0.079, 0.274], and lower athlete engagement, *β* = −0.264, 95% CI [−0.370, −0.161] (Table 6 and Figure 2). Fatigue was associated with greater difficulties in emotion regulation, *β* = 0.588, 95% CI [0.500, 0.673], and lower engagement, *β* = −0.325, 95% CI [−0.446, −0.205]. Difficulties in emotion regulation were associated with lower engagement, *β* = −0.232, 95% CI [−0.351, −0.108].

**Table 6:** Primary PLS structural-model estimates.

Path	*β*	SE	95% CI	*p*	VIF	f^2^
MSHB → Fatigue	0.491	0.042	[0.408, 0.573]	< 0.001	1.000	0.318
MSHB → Difficulties in emotion regulation	0.178	0.050	[0.079, 0.274]	< 0.001	1.318	0.046
Fatigue → Difficulties in emotion regulation	0.588	0.044	[0.500, 0.673]	< 0.001	1.318	0.504
MSHB → Athlete engagement	−0.264	0.054	[−0.370, −0.161]	< 0.001	1.379	0.095
Fatigue → Athlete engagement	−0.325	0.062	[−0.446, −0.205]	< 0.001	1.982	0.101
Difficulties in emotion regulation → Athlete engagement	−0.232	0.062	[−0.351, −0.108]	< 0.001	1.922	0.053

Percentile confidence intervals used 5,000 bootstrap resamples. MSHB = maladaptive sleep-hygiene behaviors.

**Figure 2:**
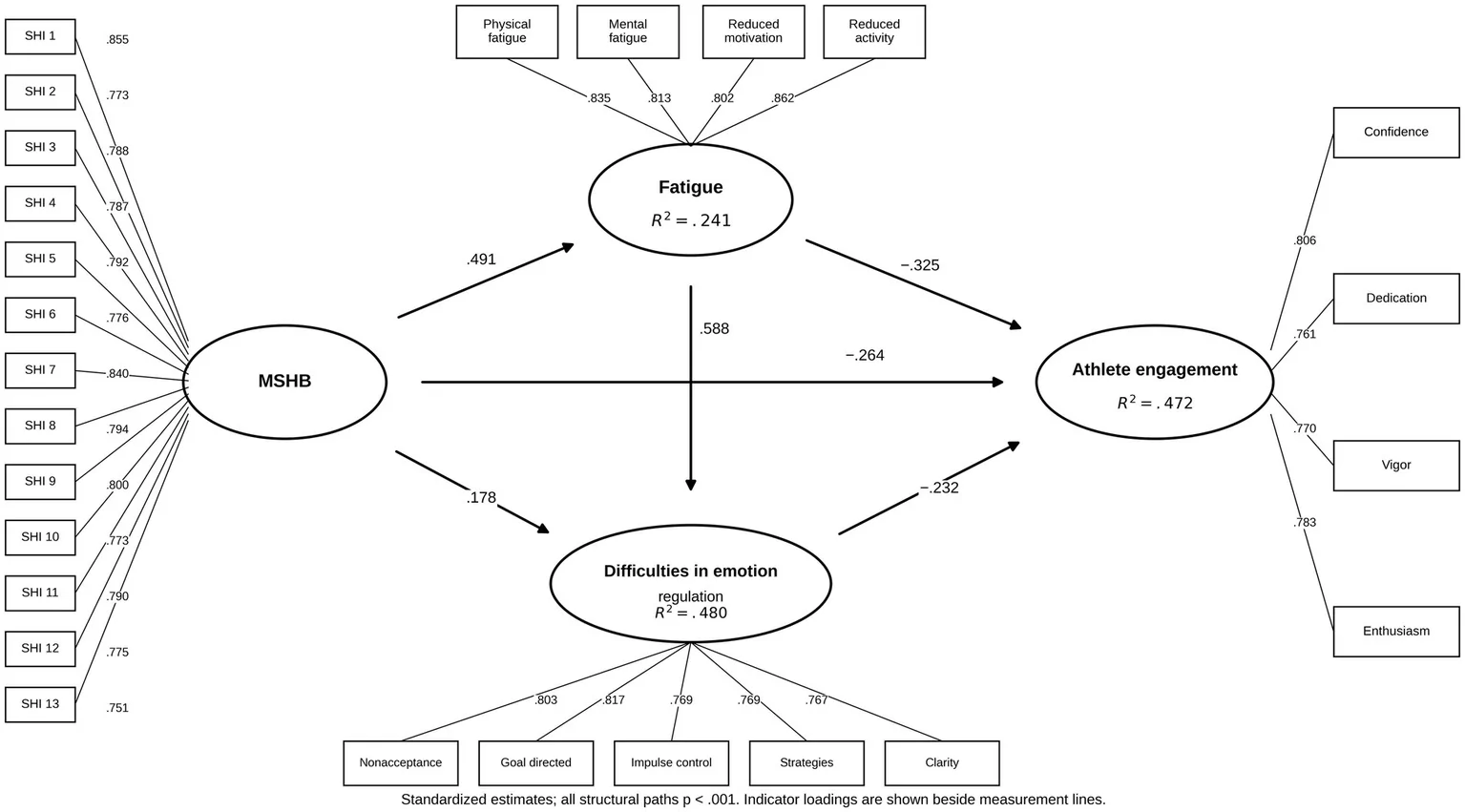
Stage-two PLS structural-model results (*N* = 317). Values on structural arrows are standardized path coefficients; values within endogenous constructs are *R*^2^. The measurement model used 13 SHI items, four fatigue domains, five emotion-regulation domains, and four engagement domains. All structural paths had *p* < 0.001.

The model explained 24.1% of fatigue variance, 48.0% of variance in difficulties in emotion regulation, and 47.2% of engagement variance. The f^2^ estimates ranged from 0.046 to 0.504. The fatigue-to-difficulties-in-emotion-regulation path had the largest incremental contribution, followed by the MSHB-to-fatigue path.

All three specific indirect associations were statistically different from zero (Table 7). The association through fatigue was *β* = −0.159, 95% CI [−0.230, −0.098]; the association through difficulties in emotion regulation was *β* = −0.041, 95% CI [−0.075, −0.014]; and the serial association in the hypothesized order was *β* = −0.067, 95% CI [−0.107, −0.031]. Their sum was *β* = −0.268, 95% CI [−0.338, −0.204]. The direct association was *β* = −0.264, and the total association was *β* = −0.532, 95% CI [−0.614, −0.448]. The combined indirect associations represented 50.4% of the total association.

**Table 7:** Bootstrap direct, indirect, total, and contrast estimates.

Effect or contrast	Estimate	SE	95% CI	*p*
MSHB → Fatigue → Engagement	−0.159	0.034	[−0.230, −0.098]	< 0.001
MSHB → Difficulties in emotion regulation → Engagement	−0.041	0.016	[−0.075, −0.014]	0.009
MSHB → Fatigue → Difficulties in emotion regulation → Engagement	−0.067	0.020	[−0.107, −0.031]	< 0.001
Total indirect association	−0.268	0.035	[−0.338, −0.204]	< 0.001
Direct association	−0.264	0.054	[−0.370, −0.161]	< 0.001
Total association	−0.532	0.043	[−0.614, −0.448]	< 0.001
Fatigue path − regulation-difficulties path	−0.118	0.042	[−0.203, −0.041]	0.005
Fatigue path − serial path	−0.092	0.045	[−0.184, −0.007]	0.041
Serial path − regulation-difficulties path	−0.026	0.019	[−0.067, 0.006]	0.169

Confidence intervals used 5,000 percentile bootstrap resamples. Contrasts compare signed indirect estimates; a negative value indicates a larger negative association for the first named path.

The fatigue-specific association represented 59.5% of the total indirect association, compared with 15.4% for the difficulties-in-emotion-regulation association and 25.0% for the serial association. The fatigue-specific association was larger in absolute magnitude than the difficulties-in-emotion-regulation association, contrast = −0.118, 95% CI [−0.203, −0.041], *p* = 0.005, and the serial association, contrast = −0.092, 95% CI [−0.184, −0.007], *p* = 0.041. The serial and difficulties-in-emotion-regulation associations did not differ, contrast = −0.026, 95% CI [−0.067, 0.006], *p* = 0.169.

### 4.3 Prediction and sensitivity analyses

PLSpredict produced positive Q^2^_predict_ values for all 13 endogenous indicators. Relative to the linear-model benchmark, the PLS model yielded lower RMSE and MAE for the four fatigue indicators and higher errors for the five emotion-regulation indicators and four engagement indicators. The model therefore showed indicator-level predictive relevance with mixed benchmark-relative performance. Indicator-level Q^2^_predict_, RMSE, and MAE results are provided in Supplementary Table S5.

Adjustment for available demographic variables produced a comparable focal path pattern. The core model included sex, age category, sport, and competitive level; the extended model also included education and years of sport experience. Every focal bootstrap confidence interval remained outside zero, and the largest absolute change from the unadjusted path estimates was less than 0.04. Training load, competition phase, injury, health, academic demands, and team context remained unmeasured alternative explanations. Primary and adjusted focal paths are compared in Supplementary Table S6.

The hypothesized-order, reverse-order, and parallel-mediator specifications reproduced the same direct, total indirect, and total associations. Their covariance-equivalent fit could not distinguish temporal ordering. MICOM supported partial measurement invariance across sex and broad sport type. No path difference was statistically significant across women and men (*p* = 0.148–0.826) or across individual racket/net and team ball sports (*p* = 0.430–0.821). These broad subgroup findings do not establish equivalence across each sport or across unmeasured teams. Detailed estimates for the alternative specifications are reported in Supplementary Table S7. MICOM results and multigroup path comparisons are presented in Supplementary Tables S8, S9, respectively.

## 5 Discussion

### 5.1 Main findings and theoretical implications

More frequent MSHB were associated with greater multidimensional fatigue, more difficulties in emotion regulation, and lower athlete engagement. The direct and combined indirect associations each accounted for approximately half of the total MSHB-engagement association, and the model explained 47.2% of engagement variance. The fatigue-specific route was the largest of the three indirect associations. These results extend engagement research beyond motivational and social conditions by locating athletes’ positive involvement within a broader pattern of daily recovery-related behavior and current functional state.

The MSHB-engagement association complements athlete-sleep research that has largely emphasized sleep quantity, quality, readiness, and performance-related outcomes (Fullagar et al., 2015; Samuels, 2008; Walsh et al., 2021). The SHI captures practices with the potential to shape sleep opportunity; it does not provide evidence about actual sleep duration, efficiency, insomnia, or nightly variability. Several explanations remain compatible with the observed association. Some behaviors may interfere with sleep opportunity, demanding schedules may produce both less consistent sleep practices and lower engagement, and broader self-management patterns may influence both. Sleep diaries and actigraphy are needed to determine how behavioral reports correspond with measured sleep.

The residual direct association indicates that fatigue and difficulties in emotion regulation did not exhaust the relationship between MSHB and engagement. Stress appraisal, attentional failures, perceived recovery, training satisfaction, coach-athlete interactions, and other lifestyle routines may contribute. Reverse association is also plausible: athletes with lower engagement may devote less attention to sleep-related routines or may encounter stressors that disturb both engagement and sleep hygiene.

### 5.2 Fatigue, emotion regulation, and model ordering

The association through fatigue accounted for the largest share of the total indirect association. This pattern fits the energetic content of engagement. Physical and mental fatigue can make training effort feel more costly, while reduced activity and motivation can accompany lower vigor, enthusiasm, and persistence. Workload and recovery research similarly shows that subjective fatigue covaries with readiness and psychological state across training periods (Curzi et al., 2024; Long et al., 2024).

Fatigue in this study should be interpreted as a broad subjective construct. Training load, illness, injury, psychological stress, travel, nutrition, and other recovery conditions can contribute to it independently of sleep hygiene. Those variables can also shape sleep practices and engagement, leaving room for residual confounding. Objective sleep and internal and external training-load data are needed to determine whether the MSHB-fatigue association reflects sleep-related effects, workload-related effects, or their combination.

Difficulties in emotion regulation contributed a smaller specific indirect association. Emotional clarity, acceptance, impulse control, access to effective strategies, and goal-directed behavior under distress are relevant when athletes manage mistakes, feedback, interpersonal tension, and selection uncertainty (Doron et al., 2025; Kucharski et al., 2018; Robazza et al., 2023). Sleep-loss research links insufficient sleep with altered cognitive control and stress-related functioning (Song et al., 2025; Vitale et al., 2021). The current findings support examining sleep-hygiene behavior and emotion regulation together in repeated-measures designs that also measure actual sleep.

The serial estimate is a model-based decomposition in the hypothesized order. Simultaneous measurement leaves temporal precedence unresolved. Equivalent fit for the hypothesized, reverse-order, and parallel specifications indicates that several directional accounts are compatible with the same covariance structure. Fatigue may complicate regulation, regulation difficulties may intensify perceived fatigue, both may reinforce one another, or common training and competitive demands may influence both. Low engagement may also precede either report.

Content proximity among the scales provides an additional boundary. Reduced motivation and activity contribute to fatigue, vigor and dedication contribute to engagement, and difficulty maintaining goal-directed behavior contributes to the DERS-16. HTMT inference and factor-model comparisons supported empirical differentiation, yet the upper fatigue-regulation HTMT limit remained close to 0.85. Shared item themes and same-source responding may therefore have increased the estimated associations.

### 5.3 Sport context and practical implications

The path pattern was similar across sex and the two broad sport categories examined, but the individual sport groups were too small for strong six-sport equivalence claims. Sleep opportunities, fatigue resistance, emotional demands, and role structures may differ across table tennis, badminton, tennis, basketball, volleyball, and football. Endurance, strength and power, combat, aquatic, rowing, and winter sports were absent. The findings are most applicable to athletes in provincial- and municipal-level teams in Sichuan with comparable characteristics.

The observed associations support joint consideration of sleep-hygiene behavior, fatigue, difficulties in emotion regulation, and engagement during observational monitoring or athlete-support assessment. A behavioral sleep measure can identify routines that warrant discussion or follow-up with sleep diaries and objective monitoring. Fatigue and engagement reports can provide complementary context. A single score should not be used to diagnose a sleep disorder or determine training readiness.

Intervention research offers candidate strategies without establishing effects on engagement in the current sample. Sleep-optimization work in football and combined sleep-hygiene and mindfulness work in youth tennis have reported benefits for selected sleep or well-being outcomes (Emami et al., 2025; Lever et al., 2021). Coordinated management among athletes, coaches, medical staff, and sleep specialists is also recommended in the wider literature (Driller et al., 2025). Whether sleep education, schedule modification, recovery planning, training-load adjustment, or emotion-regulation training improves athlete engagement requires prospective evaluation.

### 5.4 Strengths, limitations, and future research

Strengths include representation of six sports, a near-balanced sex distribution, explicit higher-order measurement specification, bootstrap confidence intervals, HTMT inference, confirmatory factor comparisons, common-method diagnostics, out-of-sample prediction, covariate adjustment, alternative-order models, and multigroup analyses. The relative contribution of each indirect association was quantified and formally compared.

Several features limit internal validity. All focal variables were self-reported at one occasion, leaving common-method inflation, reverse association, reciprocal influence, and residual confounding plausible. Training load, competition phase, professional or student-athlete status, selection pressure, injury, illness, nutrition, travel, academic burden, ambient temperature, and team context were unavailable. Team identifiers were absent, preventing adjustment for within-team dependence.

Construct validity is bounded by the instruments. The SHI measured reported sleep-hygiene behavior, the MFI measured multidimensional fatigue, and competitive performance was outside the outcome domain. The Chinese MFI was developed in military medical personnel, whereas the SHI, DERS-16, and AEQ were language-adapted for the survey. Sample-specific reliability and factor results support their use here, while replication across Chinese athlete populations, sports, competitive levels, and seasons remains necessary. Content adjacency among fatigue, regulation difficulties, and engagement should remain part of the interpretation.

External validity is limited by convenience sampling within one province, the restricted set of ball and racket/net sports, and the absence of a denominator for all eligible athletes. Provincial and municipal affiliation does not map directly onto an international elite classification or professional status. Exact age was collected categorically, and criterion-specific exclusion counts were unavailable. The estimates should therefore remain bounded to the sampled setting.

Future studies should standardize assessments within preseason, midseason, and end-of-season periods or use intensive daily designs. Objective sleep duration, timing, efficiency, and variability can be combined with sleep-hygiene reports, internal and external training load, illness and injury status, nutrition, travel, academic demands, competition schedules, and team identifiers. Multilevel longitudinal models could then separate within-athlete change from stable between-athlete differences and account for athletes nested within teams. Prospective trials could test whether changing sleep-hygiene practices changes measured sleep and whether changes in fatigue, regulation, or engagement follow.

## 6 Conclusion

Among 317 adult athletes from provincial- and municipal-level teams in Sichuan Province, more frequent maladaptive sleep-hygiene behaviors were associated with greater multidimensional fatigue, more difficulties in emotion regulation, and lower athlete engagement. The data supported specific indirect associations through fatigue and difficulties in emotion regulation and a serial indirect association in the hypothesized order; fatigue accounted for the largest specific route. Single-occasion self-report data cannot establish actual sleep disturbance, temporal precedence, causal mechanisms, or intervention effects. Repeated-measures research incorporating objective sleep, training-load, health, and competition-context indicators is needed.

## Data Availability

The original contributions presented in the study are included in the article/Supplementary material, further inquiries can be directed to the corresponding author/s.
