# Supplementary material for: Cross-sectional associations of maladaptive sleep-hygiene behaviors with athlete engagement among provincial- and municipal-level athletes in Sichuan, China
Source: Front Psychol. 2026 Sep 23;17:1914692. doi: 10.3389/fpsyg.2026.1914692 (PMC13642691; doi:10.3389/fpsyg.2026.1914692)
Supplement: Supplementary file 1 [file Table_1.DOCX]

**Supplementary analyses**

**Cross-Sectional Associations of Maladaptive Sleep-Hygiene Behaviors with Athlete Engagement among Provincial- and Municipal-Level Athletes in Sichuan, China**

**Abbreviations:** AEQ = Athlete Engagement Questionnaire; DERS-16 = 16-item Difficulties in Emotion Regulation Scale; HTMT = heterotrait-monotrait ratio; LM = linear model; MFI = Multidimensional Fatigue Inventory; MICOM = measurement invariance of composites; MSHB = maladaptive sleep-hygiene behaviors; PLS-SEM = partial least squares structural equation modeling; SHI = Sleep Hygiene Index.

All supplementary analyses used the final 317-case analytic dataset, which contained no missing values. The tables extend the main-text results by presenting domain-level, indicator-level, predictive, covariate, alternative-model, and subgroup details.

**Supplementary Table S1**

*Domain-level descriptive statistics and internal consistency for the multidimensional measures.*

| **Construct** | **Domain** | **Items** | **M** | **SD** | **Cronbach’s α** |
| --- | --- | --- | --- | --- | --- |
| Fatigue | Physical fatigue | 10 | 2.62 | .97 | .941 |
| Fatigue | Mental fatigue | 4 | 2.59 | .95 | .851 |
| Fatigue | Reduced activity | 3 | 2.37 | 1.00 | .822 |
| Fatigue | Reduced motivation | 3 | 2.61 | .96 | .773 |
| Difficulties in emotion regulation | Nonacceptance | 3 | 2.78 | 1.05 | .816 |
| Difficulties in emotion regulation | Goal-directed behavior | 3 | 2.50 | 1.05 | .863 |
| Difficulties in emotion regulation | Impulse control | 3 | 2.65 | .91 | .812 |
| Difficulties in emotion regulation | Strategies | 5 | 2.54 | .86 | .846 |
| Difficulties in emotion regulation | Clarity | 2 | 2.50 | 1.14 | .768 |
| Athlete engagement | Confidence | 4 | 3.57 | .93 | .850 |
| Athlete engagement | Dedication | 4 | 3.44 | 1.01 | .820 |
| Athlete engagement | Vigor | 4 | 3.42 | .95 | .817 |
| Athlete engagement | Enthusiasm | 4 | 3.64 | .76 | .791 |

*Note. Higher scores indicate greater fatigue, greater difficulties in emotion regulation, or stronger athlete engagement, respectively. Domain means served as reflective indicators of the corresponding higher-order constructs. The MFI item-to-variable crosswalk, domain membership, and keying rules are documented in the accompanying Data and Code archive.*

**Supplementary Table S2**

*Standardized stage-two outer loadings and 95% bootstrap confidence intervals.*

| **Construct** | **Indicator** | **Loading** | **SE** | **95% CI** |
| --- | --- | --- | --- | --- |
| MSHB | SHI 1 | .855 | .014 | [.824, .881] |
| MSHB | SHI 2 | .773 | .023 | [.723, .816] |
| MSHB | SHI 3 | .788 | .019 | [.750, .823] |
| MSHB | SHI 4 | .787 | .021 | [.743, .827] |
| MSHB | SHI 5 | .792 | .021 | [.750, .831] |
| MSHB | SHI 6 | .776 | .024 | [.725, .819] |
| MSHB | SHI 7 | .840 | .017 | [.805, .872] |
| MSHB | SHI 8 | .794 | .020 | [.753, .832] |
| MSHB | SHI 9 | .800 | .020 | [.757, .837] |
| MSHB | SHI 10 | .773 | .024 | [.722, .817] |
| MSHB | SHI 11 | .790 | .023 | [.742, .832] |
| MSHB | SHI 12 | .775 | .022 | [.728, .816] |
| MSHB | SHI 13 | .751 | .021 | [.708, .790] |
| Fatigue | Physical fatigue | .835 | .018 | [.799, .866] |
| Fatigue | Mental fatigue | .813 | .019 | [.772, .846] |
| Fatigue | Reduced activity | .862 | .015 | [.830, .888] |
| Fatigue | Reduced motivation | .802 | .021 | [.759, .839] |
| Difficulties in emotion regulation | Nonacceptance | .803 | .019 | [.763, .838] |
| Difficulties in emotion regulation | Goal-directed behavior | .817 | .019 | [.777, .851] |
| Difficulties in emotion regulation | Impulse control | .769 | .024 | [.716, .813] |
| Difficulties in emotion regulation | Strategies | .769 | .028 | [.709, .818] |
| Difficulties in emotion regulation | Clarity | .767 | .026 | [.712, .813] |
| Athlete engagement | Confidence | .806 | .019 | [.765, .843] |
| Athlete engagement | Dedication | .761 | .027 | [.702, .808] |
| Athlete engagement | Vigor | .770 | .027 | [.712, .819] |
| Athlete engagement | Enthusiasm | .783 | .025 | [.728, .828] |

*Note.* Confidence intervals were obtained from 5,000 percentile bootstrap resamples. All intervals excluded zero.

**Supplementary Table S3**

*Statistical diagnostics for common-method variance.*

| **Diagnostic** | **Result** | **Interpretation** |
| --- | --- | --- |
| Harman unrotated principal-component analysis | 12 components with eigenvalues > 1; first component = 32.54%; retained cumulative variance = 66.73% | No dominant first component |
| One-factor confirmatory factor analysis | Robust χ²(299) = 1335.53; CFI = .767; TLI = .747; RMSEA = .105, 90% CI [.099, .110]; SRMR = .105 | Poor one-factor fit |
| Full-collinearity VIF | MSHB = 1.511; fatigue = 2.181; regulation difficulties = 2.024; engagement = 1.894 | All values < 3.3 |

*Note.* The diagnostics assess whether a dominant common source is evident. Same-occasion self-report measurement remains susceptible to shared-method variance.

**Supplementary Table S4**

*Sensitivity analysis for DERS-16 score weighting.*

**Panel A. Score comparison**

| **Score** | **M** | **SD** | **Correlation with equal-domain score** | **Maximum absolute case-level difference** |
| --- | --- | --- | --- | --- |
| Equal-domain score | 2.592 | .788 | — | — |
| Item-weighted 16-item mean | 2.591 | .768 | .993 | .315 |

**Panel B. Standardized composite-score path estimates**

| **Path** | **Equal-domain DERS score** | **Item-weighted DERS score** | **Difference** |
| --- | --- | --- | --- |
| MSHB → Fatigue | .489 | .489 | .000 |
| MSHB → Difficulties in emotion regulation | .174 | .183 | .009 |
| Fatigue → Difficulties in emotion regulation | .588 | .581 | −.007 |
| MSHB → Athlete engagement | −.264 | −.262 | .001 |
| Fatigue → Athlete engagement | −.323 | −.327 | −.004 |
| Difficulties in emotion regulation → Athlete engagement | −.232 | −.227 | .004 |

*Note.* Panel B is a standardized composite-score sensitivity analysis. Difference = item-weighted estimate minus equal-domain estimate. The largest absolute path change was .009, and the substantive pattern was unchanged.

**Supplementary Table S5**

*Indicator-level PLSpredict results.*

| **Construct** | **Indicator** | **Q²_predict** | **PLS RMSE** | **LM RMSE** | **PLS MAE** | **LM MAE** |
| --- | --- | --- | --- | --- | --- | --- |
| Fatigue | Physical fatigue | .170 | .915 | .945 | .785 | .809 |
| Fatigue | Mental fatigue | .167 | .917 | .930 | .767 | .777 |
| Fatigue | Reduced motivation | .145 | .930 | .952 | .773 | .787 |
| Fatigue | Reduced activity | .159 | .922 | .952 | .759 | .784 |
| Difficulties in emotion regulation | Nonacceptance | .145 | .928 | .859 | .795 | .713 |
| Difficulties in emotion regulation | Goal-directed behavior | .140 | .932 | .822 | .770 | .672 |
| Difficulties in emotion regulation | Impulse control | .103 | .953 | .906 | .768 | .742 |
| Difficulties in emotion regulation | Strategies | .150 | .926 | .873 | .795 | .728 |
| Difficulties in emotion regulation | Clarity | .112 | .947 | .891 | .781 | .727 |
| Athlete engagement | Confidence | .182 | .909 | .876 | .737 | .688 |
| Athlete engagement | Dedication | .129 | .938 | .896 | .780 | .728 |
| Athlete engagement | Vigor | .188 | .905 | .882 | .755 | .711 |
| Athlete engagement | Enthusiasm | .167 | .917 | .869 | .768 | .704 |

*Note.* Ten-fold cross-validation was repeated 10 times. Positive Q²_predict values were obtained for all endogenous indicators. PLS errors were lower than the linear-model benchmark for the four fatigue indicators and higher for the five emotion-regulation and four engagement indicators.

**Supplementary Table S6**

*Primary and demographic-adjusted focal paths.*

| **Path** | **Primary β** | **Core adjusted β [95% CI]** | **Extended adjusted β [95% CI]** |
| --- | --- | --- | --- |
| MSHB → Fatigue | .491 | .472 [.375, .569] | .476 [.374, .578] |
| MSHB → Difficulties in emotion regulation | .178 | .159 [.056, .264] | .157 [.054, .265] |
| Fatigue → Difficulties in emotion regulation | .588 | .560 [.467, .653] | .563 [.466, .659] |
| MSHB → Athlete engagement | −.264 | −.244 [−.351, −.139] | −.239 [−.349, −.129] |
| Fatigue → Athlete engagement | −.325 | −.297 [−.413, −.174] | −.290 [−.410, −.167] |
| Difficulties in emotion regulation → Athlete engagement | −.232 | −.208 [−.326, −.087] | −.221 [−.340, −.098] |

*Note.* The core model adjusted for sex, age category, sport, and competitive level. The extended model additionally adjusted for education and years of sport experience. Categorical variables were dummy coded. Every adjusted confidence interval excluded zero, and no rounded absolute path change exceeded .04.

**Supplementary Table S7**

*Effect estimates across alternative cross-sectional mediator specifications.*

| **Specification** | **Direct association** | **Total indirect association** | **Total association** |
| --- | --- | --- | --- |
| Hypothesized order: fatigue → regulation difficulties | −.264 | −.268 | −.532 |
| Reverse order: regulation difficulties → fatigue | −.264 | −.268 | −.532 |
| Correlated parallel mediators | −.264 | −.268 | −.532 |

*Note.* The specifications were covariance-equivalent for the contemporaneous association pattern. Equivalent fit and identical total decompositions do not identify temporal order.

**Supplementary Table S8**

*MICOM results across sex and broad sport type.*

| **Grouping variable** | **Construct** | **Composite correlation** | **Permutation p** | **Mean-difference p** | **Variance-difference p** | **Compositional invariance** | **Conclusion** |
| --- | --- | --- | --- | --- | --- | --- | --- |
| Sex | MSHB | .999 | .132 | .057 | .062 | Yes | Full |
| Sex | Fatigue | 1.000 | .990 | .011 | .002 | Yes | Partial |
| Sex | Difficulties in emotion regulation | .999 | .120 | .004 | .633 | Yes | Partial |
| Sex | Athlete engagement | .997 | .053 | < .001 | .192 | Yes | Partial |
| Broad sport type | MSHB | 1.000 | .714 | .025 | .002 | Yes | Partial |
| Broad sport type | Fatigue | 1.000 | .709 | .004 | .302 | Yes | Partial |
| Broad sport type | Difficulties in emotion regulation | 1.000 | .653 | .006 | .757 | Yes | Partial |
| Broad sport type | Athlete engagement | .999 | .559 | < .001 | .833 | Yes | Partial |

*Note.* For sex, the groups were women (n = 156) and men (n = 161). For broad sport type, the groups were individual racket/net sports (n = 154) and team ball sports (n = 163). Compositional invariance was supported for every construct. Equality of composite means and variances was not universal, so partial rather than full invariance characterized the group comparisons overall.

**Supplementary Table S9**

*PLS multigroup path comparisons.*

| **Grouping variable** | **Path** | **Group 1 β** | **Group 2 β** | **Difference** | **Permutation p** |
| --- | --- | --- | --- | --- | --- |
| Sex | MSHB → Fatigue | .551 | .430 | .121 | .148 |
| Sex | MSHB → Difficulties in emotion regulation | .130 | .212 | −.082 | .412 |
| Sex | Fatigue → Difficulties in emotion regulation | .601 | .582 | .019 | .826 |
| Sex | MSHB → Athlete engagement | −.239 | −.300 | .061 | .583 |
| Sex | Fatigue → Athlete engagement | −.311 | −.340 | .029 | .810 |
| Sex | Difficulties in emotion regulation → Athlete engagement | −.258 | −.191 | −.068 | .589 |
| Broad sport type | MSHB → Fatigue | .499 | .465 | .035 | .681 |
| Broad sport type | MSHB → Difficulties in emotion regulation | .170 | .192 | −.022 | .821 |
| Broad sport type | Fatigue → Difficulties in emotion regulation | .602 | .557 | .045 | .610 |
| Broad sport type | MSHB → Athlete engagement | −.224 | −.311 | .087 | .430 |
| Broad sport type | Fatigue → Athlete engagement | −.292 | −.341 | .048 | .705 |
| Broad sport type | Difficulties in emotion regulation → Athlete engagement | −.257 | −.199 | −.058 | .648 |

*Note.* For sex, Group 1 = women (n = 156) and Group 2 = men (n = 161). For broad sport type, Group 1 = individual racket/net sports (n = 154) and Group 2 = team ball sports (n = 163). No path difference was statistically significant.
